# Stressful Effects of T-2 Metabolites and Defense Capability of HepG2 Cells

**DOI:** 10.3390/toxins14120841

**Published:** 2022-12-01

**Authors:** Mercedes Taroncher, Fiona Halbig, Yelko Rodríguez-Carrasco, María-José Ruiz

**Affiliations:** 1 Department of Preventive Medicine and Public Health, Food Science, Toxicology and Forensic Medicine, Faculty of Pharmacy, University of Valencia, 46100 Burjassot, Valencia, Spain; 2 Department of Pharmacy, Rheinische Friedrich-Wilhelms-Universität Bonn, 53115 Bonn, Germany

**Keywords:** T-2, metabolites, glutathione, antioxidant enzymes

## Abstract

The T-2 toxin (T-2), a mycotoxin produced by several species of *Fusarium* which belongs to group A of trichothecenes, is rapidly metabolized, and its main metabolites are HT-2, Neosolaniol (Neo), T2-triol and T2-tetraol. In this work, the antioxidant defense system of HepG2 cells against oxidative stress induced by T-2 and its metabolites was evaluated. The results obtained demonstrated that there is an overall decrease in glutathione (GSH) levels after all mycotoxins exposure. Moreover, the GSH levels and the enzymatic activities related to GSH (GPx and GST) increased with NAC pre-treatment (glutathione precursor) and decreased with BSO pre-treatment (glutathione inhibitor). The GPx activity is increased by T2-tetraol. The GST activity increased after T-2 and T2-triol exposure; however, T2-tetraol decreased its activity. Furthermore, CAT activity increased after T-2 and T2-triol; nevertheless, Neo decreased its activity. Finally, SOD activity is increased by all mycotoxins, except after T-2 exposure. So, the damage associated with oxidative stress by T-2 and its metabolites is relieved by the antioxidant enzymes system on HepG2 cells.

## 1. Introduction

Mycotoxins are toxic secondary metabolites produced under favorable conditions by fungi that grow on food and feed worldwide [1]. Mycotoxin contamination is an aggravated problem in developing countries. The most detected mycotoxins in food are aflatoxins (AFs), fumonisins (FBs), ochratoxin A (OTA), patulin (PAT), zearalenone (ZEA), and trichothecenes. Among the trichothecenes, the T-2 toxin (T-2) is the most cytotoxic compound [1,2]. The T-2 is produced by various species of *Fusarium*, such as *F. sporotichioides, F. poae*, *F. equiseti*, and *F. acuminatum*. It is detected in field crops such as wheat, maize, barley, and oats and processed grains such as malt, beer, and bread [3]. Furthermore, T-2 has also been detected in drinking water in endemic areas of China, such as Qinghai, and in traditional Chinese medicines [4,5,6]. The most common route of T-2 exposure is through ingestion, although there are other routes. Farmers may be exposed via direct contact with contaminated grain dust and hay, and it could cause organ damage to the kidney, liver, skin, brain, and gut [7,8].

The T-2 is rapidly metabolized to HT-2, Neosolaniol (Neo), T2-triol, and T2-tetraol [1,9]. Consequently, the cytotoxic mechanism of T-2 in vivo and in vitro might be partly attributable to its metabolites [10]. Previously, the cytotoxicity of T-2 metabolites was studied individually and in combinations in various in vitro studies, and it was observed that T-2 showed the highest cytotoxic effect [9]. The T-2 is a predisposing factor in the Kashin–Beck disease (KBD), a chronic endemic type of osteochondropathy that was mainly distributed from northeastern to southwestern China [4,11]. Moreover, the T-2 induces neurotoxicity, emesis, cardiovascular alterations, immunodepression, muscular weakness, ataxia anorectic responses, hepatotoxicity, and bone marrow damage [1,12,13]. The T-2 decreases the levels of antibodies, immunoglobulins as well as diverse cytokines [12]. An important toxicity of T-2 is mitochondrial toxicity. The T-2 may induce the collapse of mitochondrial membrane potential and promote the production of mitochondrial reactive oxygen species (ROS). Mu et al. suggested that alterations in mitochondrial gene expression could alter the coupling of mitochondrial oxidative phosphorylation leading to ROS generation [14].

Oxidative metabolites can be scavenged by GSH. The function of GSH may be evaluated in vitro by adding exogenous compounds capable of decreasing or increasing the GSH synthesis. For instance, N-Acetyl-cysteine (NAC) is an effective source of sulfhydryl groups in cells and a scavenger of free radicals because it may interact with ROS, so it is a precursor of GSH. On the other hand, D-L-buthionine-(S, R)-sulfoximine (BSO) is an inhibitor of γ-glutamylcysteine synthetase, the enzyme that triggers the cytosol’s production of GSH.

There are a variety of studies reporting the effects of oxidative stress damage generated by mycotoxins in different cell lines [15,16,17]. However, there are few reports in the literature concerning the effects of T-2 and its metabolites on enzymatic and non-enzymatic antioxidant defense. We selected the HepG2 cells due to it is a hepatic cell line, and the liver is the main drug-metabolizing organ and the main T-2 target organ [12,18]. In this study, the GSH levels and the antioxidant defense system (GPx, GST, SOD, and CAT activities) of HepG2 cells exposed to T-2, Neo, T2-triol, and T2-tetraol were evaluated. Moreover, to demonstrate the role of GSH in T-2 and its metabolite detoxification, the effect of pre-treatments with NAC or BSO was also assayed.

## 2. Results

### 2.1. In Vitro Cytotoxicity

The MTT assay was used to assess the cytotoxicity of T-2 and its modified forms on HepG2 cells after 24 h, and the results were published in our previous work [9]. The results demonstrated that T-2, Neo, T2-triol, and T2-tetraol exposure reduced cell viability in a manner that was dependent on both time and concentration. This decrease in cell viability is of the same order for all mycotoxins, being between 10 and 70% for T-2 and T2-tetraol and between 23 and 77% for Neo and T2-triol.

In this study, we have selected the IC_50_/2, IC_50_/4, and IC_50_/8 values for each mycotoxin obtained from the MTT assay. The values were 7.5, 15, and 30 nM for T-2, 12.5, 25, and 50 nM for Neo, 0.12, 0.25, and 0.45 µM for T2-triol and 0.45, 0.9, and 1.7 µM for T2-tetraol.

### 2.2. Intracellular ROS Generation

**Figure 1:**
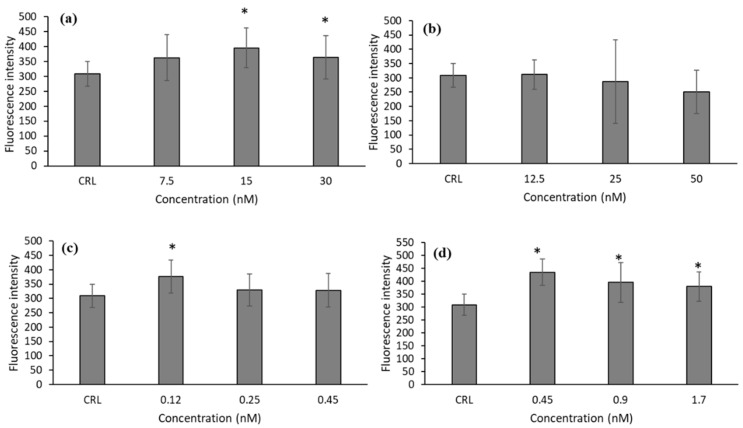
The ROS-induced fluorescence in HepG2 cells exposed to T-2 (7.5, 15, and 30 nM) (**a**), Neo (12.5, 25, and 50 nM) (**b**), T2-triol (0.12, 0.25, and 0.45 µM) (**c**) and T2-tetraol (0.45, 0.9, and 1.7 µM) (**d**) during 24 h. Results are expressed as mean ± SEM (*n* = 2). Student’s *t*-test for paired samples was used for statistical analysis of results. * *p ≤* 0.05 indicates a significant difference from the control (CRL; fresh medium).

Due to the cytotoxic effect observed of T-2 and its metabolites on HepG2 cells, we studied the generation of ROS to test if oxidative stress is one of the mechanisms by which these mycotoxins exert their cytotoxicity. As can be observed in Figure 1, there was an increase in ROS production after T-2, T2-triol, and T2-tetraol exposure. The increases in ROS production were from 17.79 to 27.92% for T-2, 21.75 for T2-triol, and 23.09 to 40.81% for T2-tetraol.

### 2.3. GSH Determination

**Figure 2:**
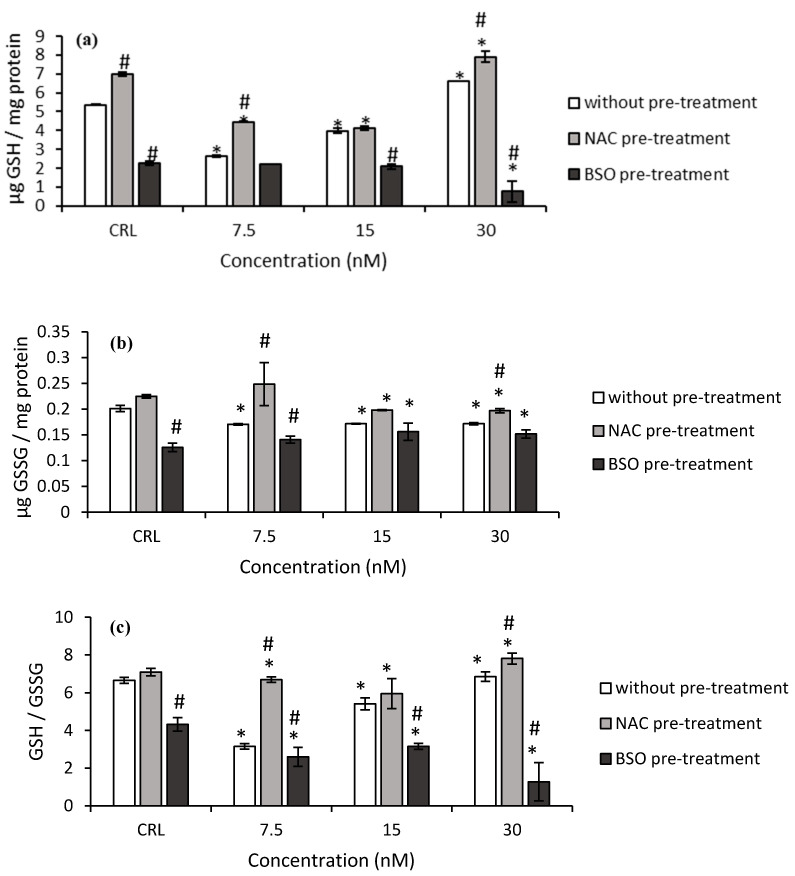
Effect of T-2 (7.5, 15, and 30 nM) with and without NAC or BSO pre-treatment on GSH levels (**a**), GSSG levels (**b**), and on the GSH/GSSG ratio (**c**) after 24 h of exposure. Data are expressed as mean values ± SEM of three independent experiments. One-way ANOVA followed by the Turkey HDS *post-hoc* test for multiple comparisons was used to analyze the difference between groups. * *p* ≤ 0.05 indicates a significant difference from the respective control (CRL; fresh medium or without pre-treatment, NAC pre-treatment, and BSO pre-treatment); # *p* ≤ 0.05 indicates a significant difference with respect to the fresh medium.

As far as the GSH determination after T-2 exposure is concerned, the GSH levels and GSH/GSSG ratio significantly decreased (*p ≤* 0.05) at 7.5 nM by 50.6 and 52.7% and at 15 nM by 25.8 and 18.7%, respectively, compared to control (Figure 2a,c). The GSSG levels also decreased at all the assayed concentrations with respect to control cells (Figure 2b).

Regarding NAC pre-treatment, a significant increase in GSH levels (30.1%) in control cells was observed with respect to control without NAC pre-treatment (Figure 2). Also, at 7.5 and 30 nM T-2, NAC pre-treatment led to an increase in GSH level (68.3 and 19.3%, respectively), GSSG content (45.7 and 14.7%, respectively), and the GSH/GSSG ratio (112.4 and 13.9%, respectively) with respect to cells in fresh medium, showing a NAC protective effect in HepG2 cells. Furthermore, an increase of GSH (13.3%) and GSH/GSSG (10%) at 30 nM was observed, corresponding with a decrease (12.3%) of GSSG levels, compared to control with NAC pre-treatment.

Concerning the BSO pre-treatment, a decrease in GSH level (57.6%), GSSG level (37.4%), and GSH/GSSG ratio (35.2%) was obtained in their own controls compared to controls without pre-treatment. In this respect, a significant decrease in GSH levels was determined at 47.2 and 88.5% at 15 and 30 nM, respectively. The GSH/GSSG ratio also decreased from 17.6 to 81.4% (Figure 2c). On the other hand, a significant decrease in GSH levels (66.6%) was also obtained after the exposure of T-2 at the highest concentration (30 nM) compared to their own control, whereas a significant decrease in GSH/GSSG ratio (from 26.9 to 70.5%) was obtained with respect to control with BSO pre-treatment.

**Figure 3:**
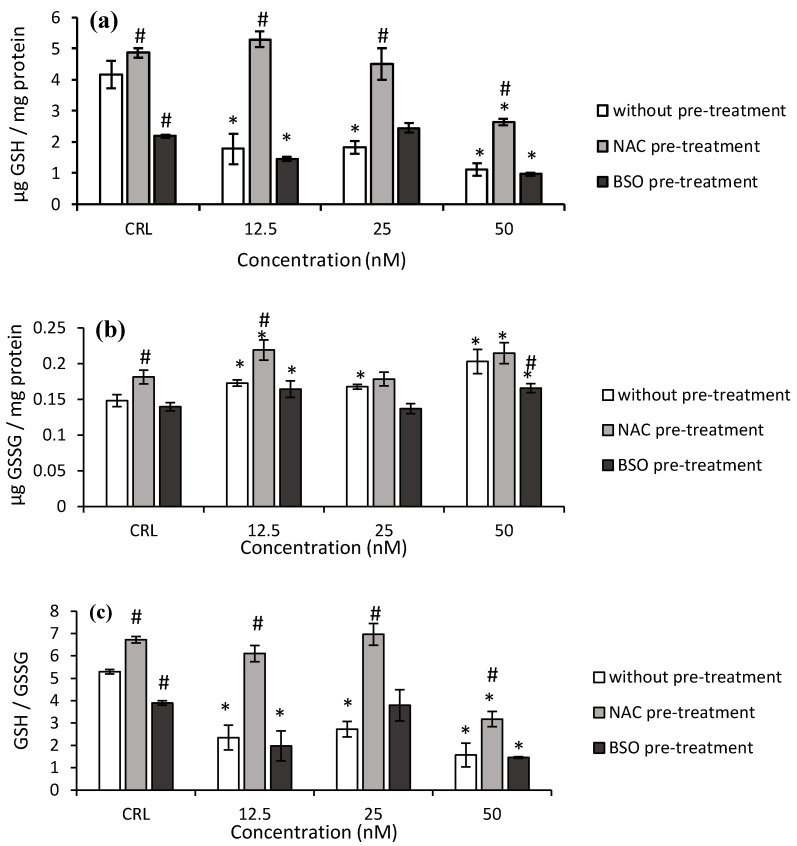
Effect of Neo (12.5, 25, and 50 nM) with and without NAC or BSO pre-treatment on GSH levels (**a**), GSSG levels (**b**), and on the GSH/GSSG ratio (**c**) after 24 h of exposure. Data are expressed as mean values ± SEM of three independent experiments. One-way ANOVA followed by the Turkey HDS *posthoc* test for multiple comparisons was used to analyze the difference between groups. * *p* ≤ 0.05 indicates a significant difference from the respective control (CRL; fresh medium or without pre-treatment, NAC pre-treatment, and BSO pre-treatment); # *p* ≤ 0.05 indicates a significant difference with respect to the fresh medium.

Regarding Neo (Figure 3), in fresh medium, the GSH levels and GSH/GSSG ratio significantly decreased from 56.2 to 73.3%, and from 48.6 to 70.4%, after Neo exposure, respectively, compared to control cells (Figure 3a,c). On the contrary, the GSSG content significantly increased from 13.2 to 37.1% (Figure 3b).

Concerning the NAC pre-treatment, an increase of GSH and GSSG levels and GSH/GSSG ratio (by 16.9, 22.4, and 26.8%, respectively) in control cells was observed with respect to control in fresh medium (Figure 3). Similarly, the pre-treatment with NAC produced an increase in GSH levels (from 137.4 to 197.6%) and GSH/GSSG ratio (from 102.3 to 159.9%) in cells exposed to Neo, with respect to cells in fresh medium, which showed a cytoprotecting effect of NAC in these cells.

With respect to BSO pre-treatment, a decrease in the GSH levels and GSH/GSSG ratio (47.4 and 26.4%, respectively) was obtained in controls compared to controls without BSO. The GSSG levels significantly decreased by 18.3% at the highest Neo concentration. Additionally, a significant decrease of GSH levels (by 32.9 and 55.9%) and GSH/GSSG ratio (by 49.4 and 62.6%) and a significant increase in GSSG levels (by 17.6 and 18.6%) were also obtained after 12.5 and 50 nM of Neo exposure, respectively, compared to BSO pre-treated controls.

**Figure 4:**
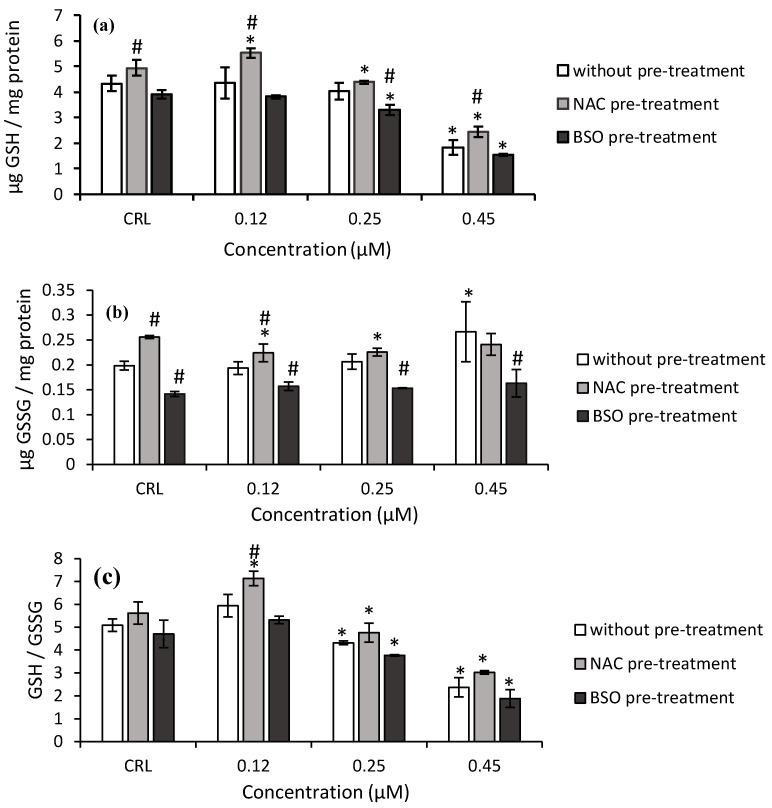
Effect of T2-triol (0.12, 0.25, and 0.45 µM) with and without NAC or BSO pre-treatment on GSH levels (**a**), GSSG levels (**b**), and on the GSH/GSSG ratio (**c**) after 24 h of exposure. Data are expressed as mean values ± SEM of three independent experiments. One-way ANOVA followed by the Turkey HDS *posthoc* test for multiple comparisons was used to analyze the difference between groups. * *p* ≤ 0.05 indicates a significant difference from the respective control (CRL; fresh medium or without pre-treatment, NAC pre-treatment, and BSO pre-treatment); # *p* ≤ 0.05 indicates a significant difference with respect to the fresh medium.

Regarding T2-triol (Figure 4), the GSH levels and GSH/GSSG ratio decreased after 0.45 µM T2-triol exposure (57.9 and 53.3%, respectively) in fresh medium, whereas the GSSG content significantly increased (34.2%) compared to control cells.

Concerning the effects of NAC pre-treatment, a significant increase of GSH and GSSG levels was determined in control cells by 14 and 28.8%, respectively, compared to controls without NAC pre-treatment (Figure 4). Additionally, NAC pre-treatment induced an increase in GSH level (from 26.8 to 34.7%), GSSG levels (15.7%), and GSH/GSSG ratio (20.1%) in cells exposed to T2-triol compared to cells in fresh medium (Figure 4).

Regarding the BSO pre-treatment, a significant decrease in the GSH levels (up to 60.3%) and GSH/GSSG ratio (up to 59.9%) were also obtained after T2-triol exposure compared to its own control. Finally, a decrease up to 38.7% of GSSG content of T2-triol was observed compared to cells without BSO pre-treatment (Figure 4b).

**Figure 5:**
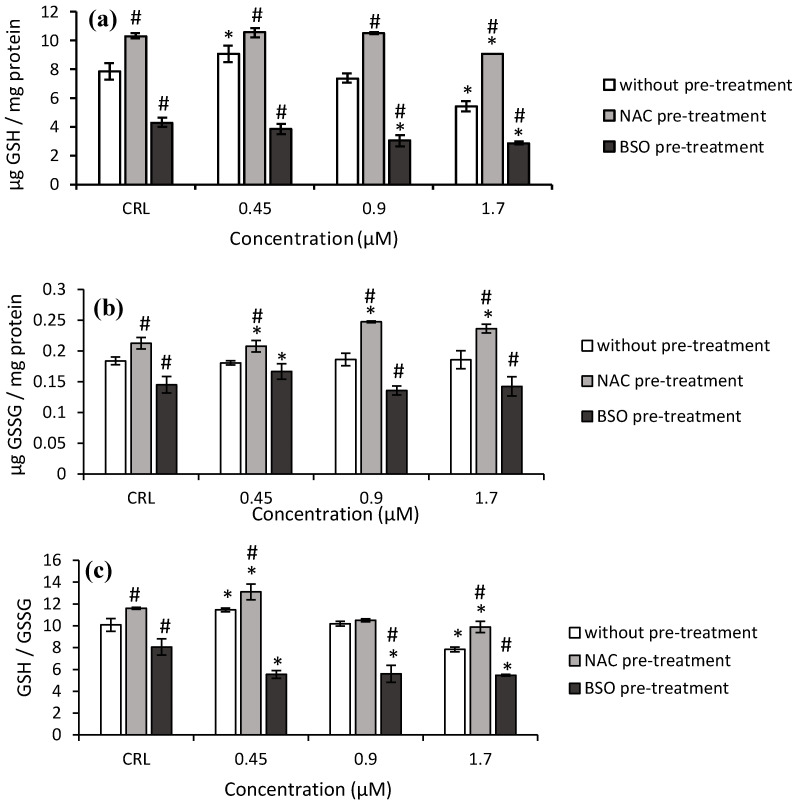
Effect of T2-tetraol (0.45, 0.9, and 1.7 µM) with and without NAC or BSO pre-treatment on GSH levels (**a**), GSSG levels (**b**), and on the GSH/GSSG ratio (**c**) after 24 h of exposure. Data are expressed as mean values ± SEM of three independent experiments. One-way ANOVA followed by the Turkey HDS *posthoc* test for multiple comparisons was used to analyze the difference between groups. * *p* ≤ 0.05 indicates a significant difference from the respective control (CRL; fresh medium or without pre-treatment, NAC pre-treatment, and BSO pre-treatment); # *p* ≤ 0.05 indicates a significant difference with respect to the fresh medium.

Similar results were obtained with T2-tetraol. The GSH levels and GSH/GSSG ratio increased (15.3 and 13.7%, respectively) at 0.45 µM and decreased (31.1 and 22.4%, respectively) at 1.7 µM with respect to the control cells. (Figure 5).

The NAC pre-treatment produced an increase in GSH levels (30.9%), GSSG levels (15.6%), and GSH/GSSG ratio (15.2%) in controls compared to controls without NAC. When cells exposed to NAC pre-treatment were compared to cells exposed to fresh medium, an increase of GSH, GSSG levels, and the GSH/GSSG ratio was observed. These values increased to 66.8%, 32.9%, and 26.4%, respectively.

Concerning the BSO pre-treatment, a significant decrease in the GSH, GSSG levels, and GSH/GSSG ratio was observed (Figure 5). The BSO pre-treatment showed a decrease from 47.1 to 58.9% of GSH levels, from 23.3 to 27.1% of GSSG levels and from 30 to 51.6% of GSH/GSSG ratio, with respect to cells without BSO. Finally, with BSO pre-treatment, a significant decrease of up to 31.9% was observed in the GSH/GSSG ratio with respect to its own control.

### 2.4. Enzymatic Activity

The GPx, GST, CAT, and SOD activities were assessed in HepG2 cells after 24 h of incubation with T-2 (7.5, 15, and 30 nM) and its modified forms, Neo (12.5, 25, and 50 nM), T2-triol (0.12, 0.25 and 0.45 µM) and T2-tetraol (0.45, 0.9 and 1.7 µM).

**Figure 6:**
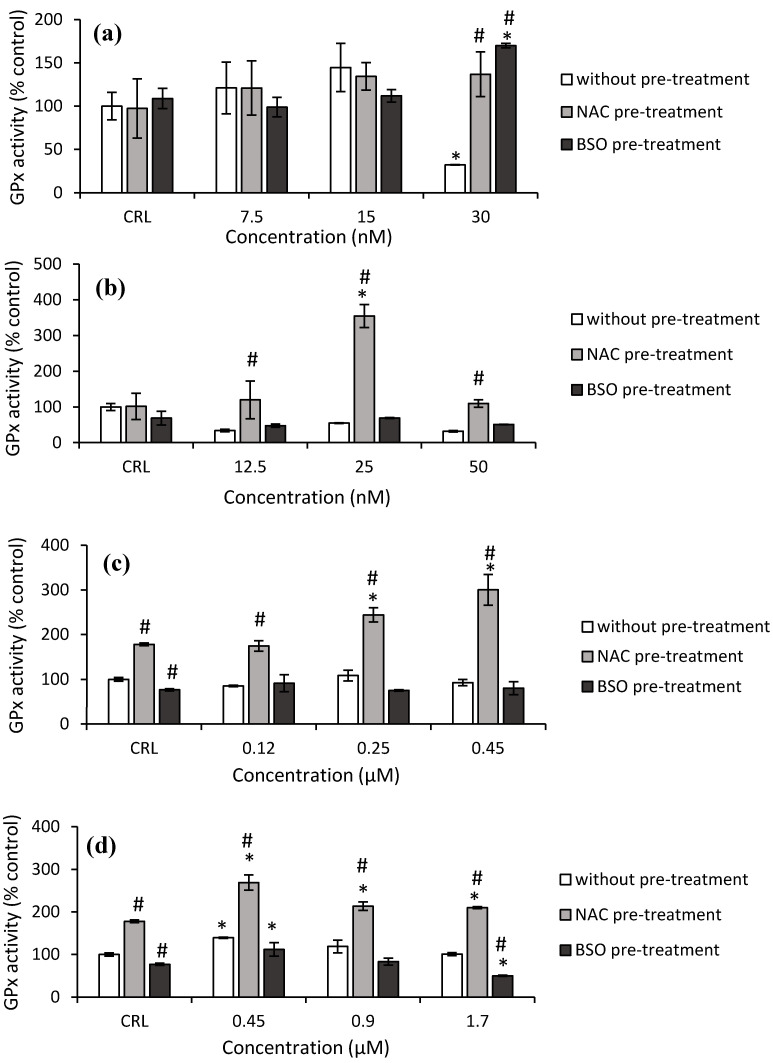
Effect of T-2 (7.5, 15 and 30 nM) (**a**), Neo (12.5, 25, 50 nM) (**b**), T2-triol (0.12, 0.25, 0.45 µM) (**c**) and T2-tetraol (0.45, 0.9, 1.7 µM) (**d**) with and without NAC or BSO pre-treatment on glutathione peroxidase activity after 24 h of exposure. Data are expressed in % of the unexposed control (CRL). In terms of μmol of NADPH oxidized/min/mg of protein, the GPx activity is expressed; mean ± SEM (*n* = 3). One-way ANOVA followed by the Turkey HDS *posthoc* test for multiple comparisons was used to analyze the difference between groups. * *p* ≤ 0.05 indicates a significant difference from the respective control (fresh medium or without pre-treatment, NAC pre-treatment, and BSO pre-treatment); # *p* ≤ 0.05 indicates a significant difference with respect to the fresh medium.

Concerning GPx activity, a significant decrease (67.9%) was obtained after 30 nM T-2 exposure in the fresh medium, as shown in Figure 6a, whereas a significant increase was observed at this concentration in cells pre-treated with NAC (326.4%) pre-treatment, compared to cells in fresh medium. Regarding to Neo exposure, an increase by 249.2% at 25 nM was determined in NAC pre-treated cells compared to its own control, while an increase (from 242.9 to 548.3%) was observed at all concentrations tested compared to cells in fresh medium (Figure 6b).

Respect to T2-triol, control NAC pre-treated increased (77.9%) the GPx activity while control BSO pre-treated significantly decreased (23.2%) with respect to fresh medium control cells. Cells NAC pre-treated increased the GPx activity from 37.2 to 68.8% compared to its own control, and it increased (from 104.5 to 223.7%) with respect to fresh medium cells (Figure 6c). Finally, regarding T2-tetraol, an increase in GPx activity of 77.9% was shown in control NAC pre-treatment compared to control without pre-treatment, while a decrease of 23.2% in control BSO pre-treatment was observed. The GPx activity increased by 39.2%, from 18 to 51.2% and by 79.8 to 108.4%, in HepG2 cells exposed to T2-tetraol in fresh medium and in cells pre-treated with NAC with respect to its own control and respect to the corresponding cells in fresh medium, respectively. On the other hand, with BSO pre-treatment, the GPx activity decreased with respect to cells in fresh medium (50.7%) and with respect to its own control (35.3%) after the highest concentration of T2-tetraol (Figure 6d).

**Figure 7:**
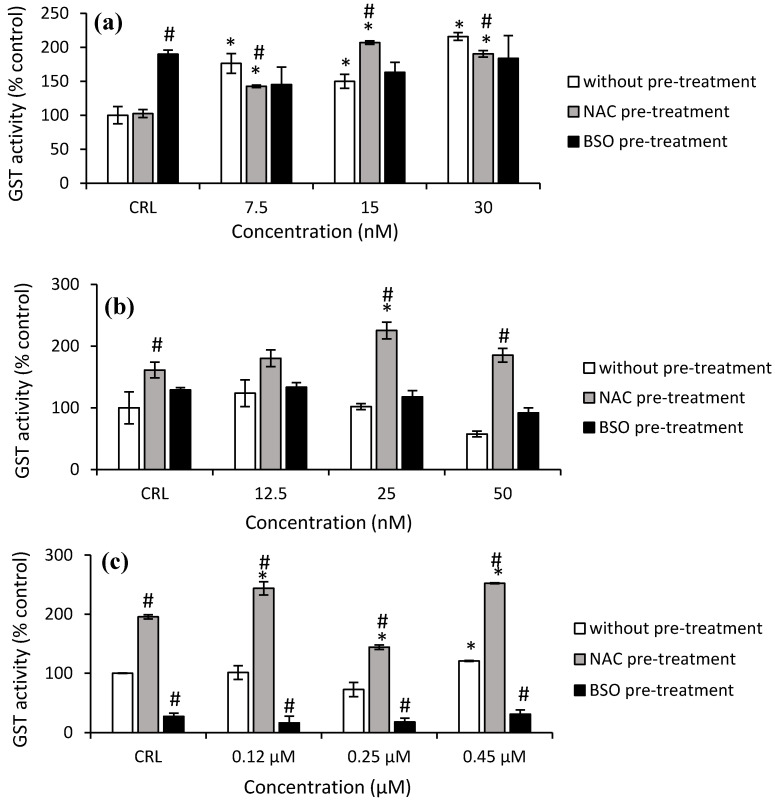

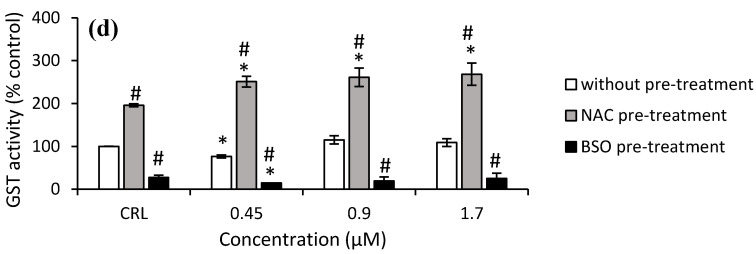
Effect of T-2 (7.5, 15 and 30 nM) (**a**), Neo (12.5, 25, 50 nM) (**b**), T2-triol (0.12, 0.25, 0.45 µM) (**c**) and T2-tetraol (0.45, 0.9, 1.7 µM) (**d**) with and without NAC or BSO pre-treatment on glutathione S-transferase activity after 24 h of exposure. Data are expressed in % of the unexposed control (CRL). In terms of mol of product formed/min/mg of protein, the GST activity is expressed; mean ± SEM (*n* = 3). One-way ANOVA followed by the Turkey HDS *posthoc* test for multiple comparisons was used to analyze the difference between groups. * *p* ≤ 0.05 indicates a significant difference from the respective control (fresh medium or without pre-treatment, NAC pre-treatment, and BSO pre-treatment); # *p* ≤ 0.05 indicates a significant difference with respect to the fresh medium.

As shown in Figure 7a, the GST activity increased significantly in HepG2 cells from 49.8% to 115.7% after T-2 exposure in the fresh medium. After NAC pre-treatment, the highest increase in GST activity was observed at 15 nM T-2 compared to its own control cells (by 102.3%) and with respect to cells in fresh medium (by 38.2%). Regarding Neo exposure, it only was observed in cells with NAC pre-treatment and led to an increase to 221.8% (50 nM) and 39.8% (25 nM) compared to cells without pre-treatment and to control with NAC pre-treatment, respectively (Figure 7b).

With regard to T2-triol exposure, a significant increase (20.9%) at 0.45 µM was shown in cells with fresh medium compared to the control. Regarding the effects of NAC pre-treatment, an increase of 95.6% in control NAC pre-treated cells compared to control no pre-treated was obtained. Moreover, an increase of GST activity after T2-triol exposure up to 29% compared to control cells with NAC pre-treatment was observed as well as an increase from 97.5 to 140.6% compared to cells without pre-treatment. Conversely, a decrease of 72.6% in control BSO pre-treated compared to control cells not pre-treated was shown. Similarly, a decrease of up to 83.7% after T2-triol exposure was observed compared to cells without BSO pre-treatment (Figure 7c). Finally, regarding T2-tetraol exposure, an increase of 95.6% was shown in control cells NAC pre-treated compared to control no pre-treated, whereas BSO pre-treated showed a decrease of 72.6%. Furthermore, a significant increase was obtained at all concentrations tested in cells pre-treated with NAC with respect to its own control cells (from 28.3 to 37.2%) and also to the corresponding cells in fresh medium (from 146.5 to 227.7%). On the other hand, the GST activity decreased in cells BSO pre-treated exposed to 0.45 µM by 45.8% compared to control BSO pre-treated, whilst a decrease at all concentrations tested in cells pre-treated with BSO with respect to cells without pre-treatment from 80.6 to 76.9% was also observed (Figure 7d).

**Figure 8:**
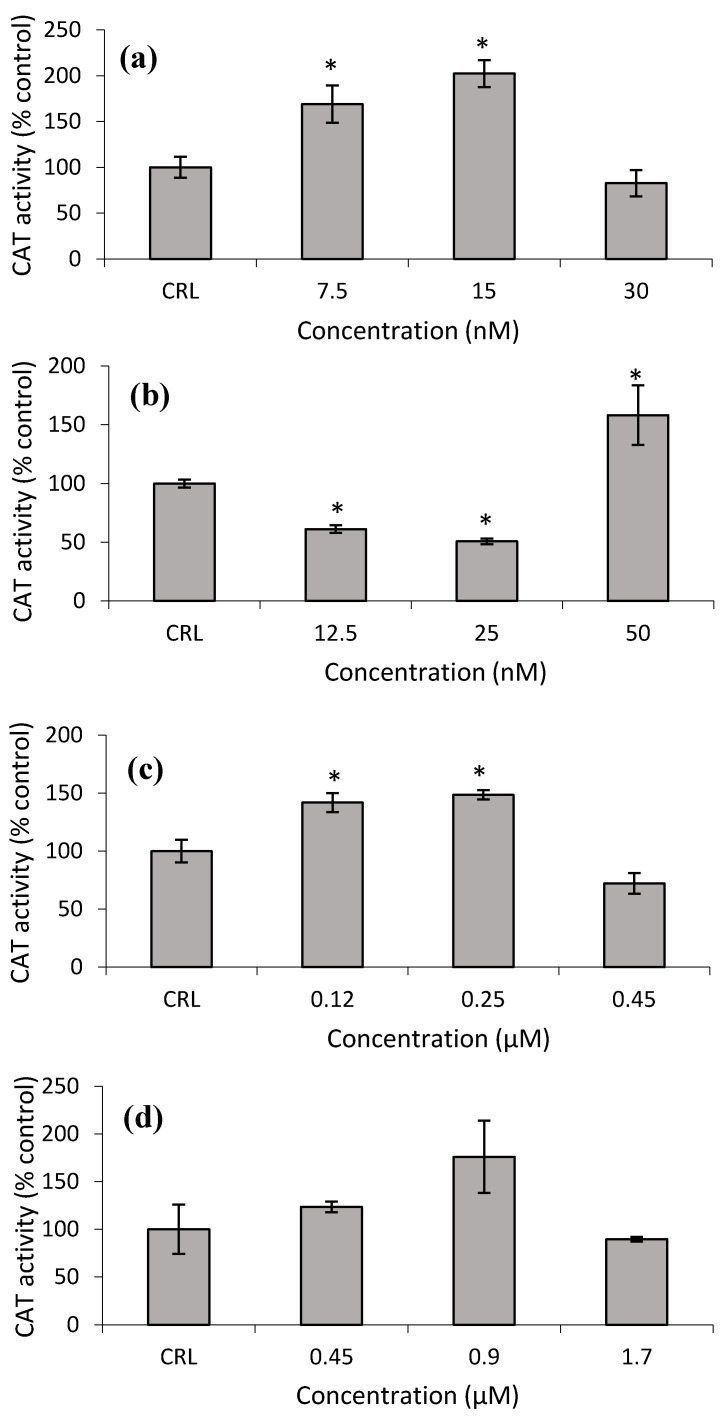
Effect of T-2 (7.5, 15 and 30 nM) (**a**), Neo (12.5, 25, 50 nM) (**b**), T2-triol (0.12, 0.25, 0.45 µM) (**c**) and T2-tetraol (0.45, 0.9, 1.7 µM) (**d**) on catalase activity after 24 h of exposure. Data are expressed in % of the unexposed control (CRL). The CAT activity is expressed as µmol of H_2_O_2_/min/mg of protein; mean ± SEM (*n* = 3). Student’s *t*-test for paired samples was used for statistical analysis of results. * *p* ≤ 0.05 indicates a significant difference with respect to the control.

Figure 8a shows that the CAT activity increased at the lower T-2 and T2-triol concentrations from 68.9 to 102.2% and from 41.9 to 48.6%, respectively, with respect to control cells (Figure 8c). Conversely, at the lower Neo concentrations, the CAT activity significantly decreased from 38.9 to 49.3%, while an increase of 58.2% was detected at the highest Neo concentration with respect to control (Figure 8b,d).

**Figure 9:**
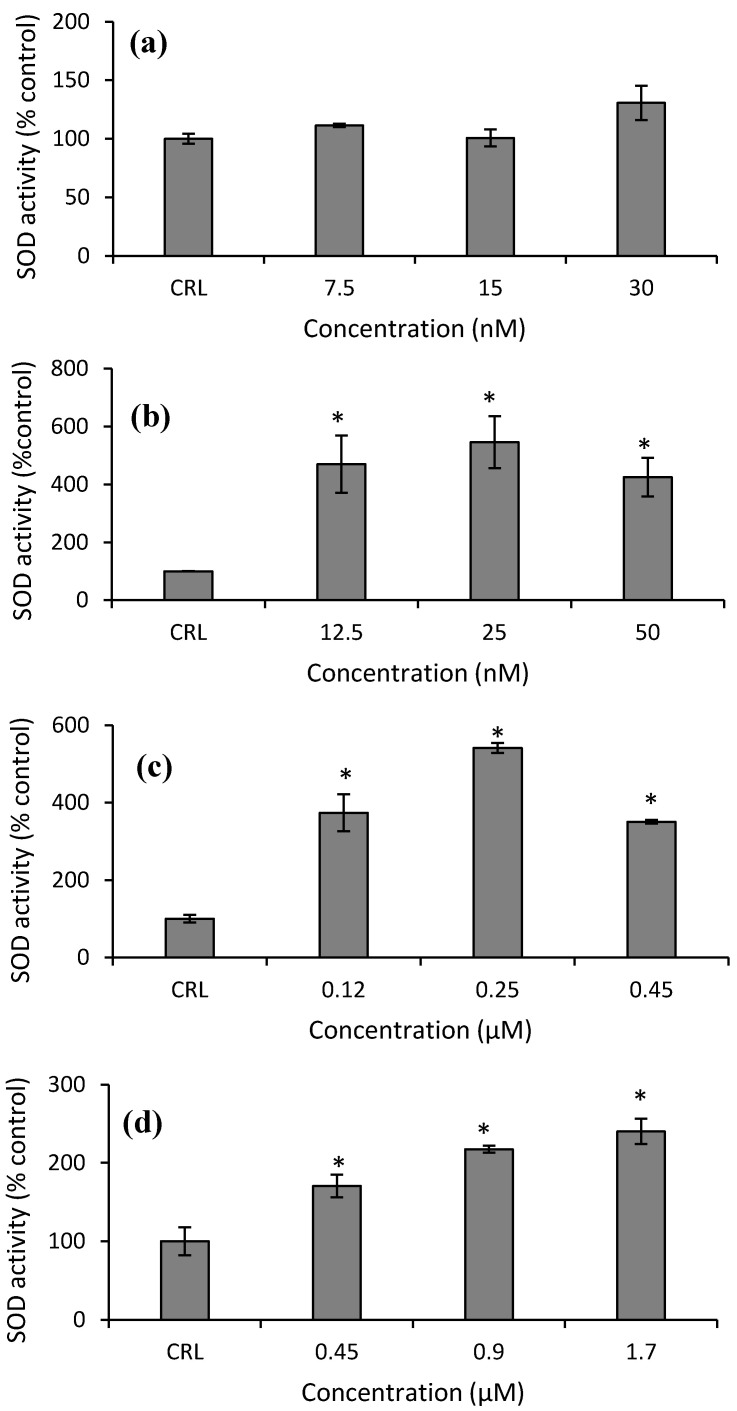
Effect of T-2 (7.5, 15 and 30 nM) (**a**), Neo (12.5, 25, 50 nM) (**b**), T2-triol (0.12, 0.25, 0.45 µM) (**c**) and T2-tetraol (0.45, 0.9, 1.7 µM) (**d**) on superoxide dismutase activity after 24 h of exposure. Data are expressed in % of the unexposed control (CRL); mean ± SEM (*n* = 3). Student’s *t*-test for paired samples was used for statistical analysis of results. * *p* ≤ 0.05 indicates a significant difference with respect to the control.

Finally, with respect to SOD activity in HepG2 cells, an increase at all concentrations tested of Neo (from 325.3 to 445.9%), T2-triol (from 250.65 to 441.2%), and T2-tetraol (from 70.4 to 140.2%) compared to their respective controls was obtained (Figure 9b–d).

## 3. Discussion

This study showed that in HepG2 cells, T-2, T2-triol, and T2-tetraol triggered an increase of intracellular ROS levels after 24 h of exposure (Figure 1), demonstrating that oxidative stress plays a role in cellular toxicity.

The induction of oxidative stress by T-2 has been previously demonstrated by other authors [3,10,11,19,20,21,22,23,24,25,26,27]. The results obtained for T-2 in HepG2 cells grown in medium showed a decrease in GSH levels at 7.5 and 15 nM compared to control cells. However, intracellular GSH increases at 30 nM and it may be to keep the cells in a reduced state and to avoid oxidative stress to high doses (Figure 1a and Figure 2a).

In our results, correlated with the decrease in GSH levels, an increase in GST activity was observed. However, GPx activity remained unchanged (Figure 6a and Figure 7a). According to Gouze et al. [28], the 12,13-epoxide group, which characterizes all epoxy-trichothecene toxins, could be a substrate for GST conjugation, as GST is active in the epoxides group. These authors demonstrated that deoxynivalenol (DON) trichothecene has the ability to conjugate GST, as DON is a substrate of GST [28]. Therefore, in this work, the increase of GST enzymatic activity after T-2 exposure leads us to speculate that this mycotoxin could be a substrate for GST due to the structural similarity of both trichothecenes. So, the GST-mediated conjugation could represent a detoxification pathway in protecting cells from injury by T-2. On the other hand, GPx activity in HepG2 is not the main implicated enzyme in detoxifying against the cytotoxicity of T-2. Also, it is possible that GPx inactivates itself by the decrease of its own substrate. Similarly, SOD activity remains unchanged after T-2 exposure. Nevertheless, CAT activity increased (Figure 8a and Figure 9a). This can be due to the fact that T-2 increases ROS (Figure 1a), including H_2_O_2_, and it is transformed rapidly to H_2_O and O_2_ by CAT. Thus, our results suggest that the main enzymes involved in detoxifying T-2 in HepG2 cells are GST and CAT. Therefore, GST activity remains high since this enzyme is important to protect against oxidative stress to which the HepG2 cells are submitted, as shown by the equally high CAT activity and GSH content at the highest T-2 concentration tested.

Similar results to our SOD activity were reported in HepG2 cells exposed to OTA, in Hek-293 cells after DON exposure, and in SH-SY5Y cells after beauvericin (BEA), α-ZEL, and β-ZEL exposure. These authors demonstrated that the activity of SOD tended to the control levels, that are, cells exposed to culture medium only, without mycotoxins [29,30,31]. Regarding enzymatic activities in cell culture exposed to mycotoxin, there is a variety of results. According to our results, Yang et al. [10,18] observed an increase in CAT, SOD, and GPx activity after T-2 exposure (from 10 to 100 nM) in broiler hepatocytes. Conversely, Li et al. [19] and Zhang et al. [3] reported a decrease in CAT activity after T-2 exposure to porcine kidney and Leydig cells. The increase of CAT, GPx, and SOD activities suggests that in hepatocytes, the T-2 increases ROS which is catalyzed by SOD, and the high levels of H_2_O_2_ as a result of the reaction are detoxified by CAT and GPx, decreasing the GSH levels. On the contrary, the SOD, GPx, and CAT enzymes do not seem to be the main enzymes responsible for the detoxification in Leydig or porcine kidney cells in response to T-2 exposure. So, these enzymes are not activated by the cells to prevent oxidative stress.

The mechanism of toxicity of T-2 modified forms is the innovative part of this study, as little or no information is available. Regarding Neo, a significant decrease in GSH and an increase in GSSG levels at all concentrations tested were determined in HepG2 cells (Figure 3a,b). These results are similar to the effects of T-2 in this work. Nevertheless, the depletion in GSH levels seems unrelated to its involvement through the activity of GPx, due to its activity remaining unchanged (Figure 6b and Figure 7b). So, the enzymatic cell system not related to GSH is the most important enzymatic defense system in HepG2 cells exposed to Neo. The increase in SOD activity has also been observed in GH3 and broiler hepatocytes [10,11,18] after T-2 exposure. Other authors reported differences in the results of the CAT activity depending on the range of concentration tested in different cell types exposed to T-2 [3,19,29,32]. However, no data were reported about Neo.

A significant decrease in GSH and an increase in GSSG levels at the highest concentration of T2-triol (0.45 µM) tested were found in HepG2 cells (Figure 4a,b). Similar effects were observed with ZEA and its metabolites on CHO-K1, HepG2, and SH-SY5Y cells [29,32,33], BEA in CHO-K1 cells [34], and OTA in HepG2 cells [31]. The decrease in GSH levels is accompanied by an increase in GST at 0.45µM. However, GPx activity remains unchanged (Figure 6c and Figure 7c). This indicates that the T2-triol detoxifying enzyme is mainly GST.

On the other hand, the increase of ROS after T2-triol exposure (Figure 1c) provide the stimulus for increasing the CAT and SOD activities at all concentrations (Figure 8c and Figure 9c). Much research has been carried out to assess the impact of different mycotoxins on the antioxidant defense system by different authors. However, as far as the author´s knowledge extends, no data about the effects of T-2 metabolites on the enzymatic antioxidant protective system has yet to be found in the literature.

On HepG2 cells, intracellular GSH increased immediately at the lowest concentration (0.45 µM) of T2-tetraol, acting as a major thiol-disulfide redox buffer as a response to oxidative damage to keep the cells in a reduced state (Figure 5a). However, with the higher concentration (1.7 µM), GSH is depleted due to high levels of oxidative stress. On the other hand, the increase in GSH content was accompanied by an increase in GPx activity at 0.45 µM. Nonetheless, the GST activity decreased (Figure 6d and Figure 7d). So, regarding T2-tetraol, the main enzymatic detoxifying mechanism involved was GPx. On the other hand, the oxidizing compounds produced by T2-tetraol were catalyzed by the SOD enzyme because their activity increased at all concentrations tested. The increase of ROS after T2-tretraol exposure (Figure 1d) leads to GSH action (Figure 5a) and also to SOD activity (Figure 9d). On the contrary, the T2-tetraol did not stimulate CAT activity, so we can conclude that T2-tetraol was detoxified by GPx and SOD enzymatic activities (Figure 8d and Figure 9d). In previous work, we demonstrated that the T-2 is converted by hydrolysis to Neo and T2-triol, but all three metabolites end up as T2-tetraol [9], and it is known that the GPx is the most important enzyme for the extra peroxisomal inactivation of H_2_O_2_, particularly in liver cells. This is related to our results, which show that in HepG2 cells, the final metabolite T2-tetraol is detoxified mainly by the GPx enzyme. According to our results, the increase in GPx and SOD enzymatic activities was previously reported for T-2 [10,11,18] and for DON [35]. Furthermore, similar to our results, the tendency of CAT to basal rate levels [17,30,31] and the decrease in GST [17,29] was previously observed after OTA, DON, and STE exposure in cell cultures.

The role of the pre-treatment with NAC and BSO, a GSH promoter, and a GSH depletory, respectively, was also evaluated. For T-2, Neo, T2-triol, and T2-tetraol, the GSH levels were increased in cells with NAC pre-treatment because the GSH is involved in the cellular defense mechanism when HepG2 cells were exposed to these mycotoxins. It was evidenced by the increase in GPx and GST activities after NAC pre-treatment in all mycotoxin treatments. We determined that pre-treatment with the antioxidant NAC suppressed oxidative damage induced by T-2 and its metabolites on HepG2 cells. On the contrary, overall, BSO showed a significant decrease in GSH levels in cells exposed to T-2, T2-triol, and T2-tetraol. However, no effects were observed after Neo exposure compared to cells without BSO pre-treatment. This can be because the GPx and GST enzymes (depending on GSH levels) are not involved in the Neo detoxification mechanism. In fact, no changes were observed in GPx (after Neo and T2-triol exposure) and GST (after Neo exposure) activities after BSO pre-treatment compared to cells without pre-treatment. On the contrary, GST activity after BSO exposure is highly suppressed because the GSH levels decrease, and GST is a GSH-related enzyme. Similar results related to the antioxidant defense system after NAC and BSO pre-treatment have already been described on HepG2 [36,37] and SH-SY5Y cells [17]. According to these authors, the NAC blocks ROS induced by STE on SH-SY5Y cells because the GSH levels and GPx and GST activity increased compared to cells without NAC pre-treatment [17]. The same cells have more predisposition to oxidative damage induced by STE after BSO pre-treatment because GSH levels decreased in cells treated with STE at all concentrations tested further than in cells without pre-treatment, leading to a decrease in GPx and GST activities. The results obtained in this work are similar to those obtained by these authors and confirm that GSH was involved in the defense mechanism of the HepG2 cells after trichothecenes exposure.

## 4. Conclusions

In conclusion, our results suggest that T-2, Neo, T2-triol and T2-tetraol lead to the actuation of the GSH redox system in HepG2 cells because the decrease of intracellular GSH levels was observed after all mycotoxins and the stimulation of GSH dependent enzyme system. So, the GSH depletion confirmed oxidative stress as an underlying mechanism involved in the cytotoxicity of T-2 and its metabolites. Nevertheless, regarding antioxidant enzymes, each mycotoxin acts in a different way. Furthermore, the GSH levels and the enzymatic activities related to GSH increased in NAC pre-treated and decreased in BSO pre-treated cells. So, the damage associated with oxidative stress by T-2 and its metabolites is relieved by the antioxidant enzymes system on HepG2 cells.

## 5. Materials and Methods

### 5.1. Reagents

The substances utilized for cell culture and reagent-grade chemicals include Dulbecco’s Modified Eagle’s Medium (DMEM), streptomycin, penicillin, phosphate-buffered saline (PBS), trypsin/EDTA solutions, newborn calf serum (NBCS), sodium azide (NaN_3_), β–nicotinamide adenine dinucleotide phosphate (β-NADPH), GSH, GSSG, NAC, BSO, N-ethylmaleimide (NEM), o-phtaldialdehyde (OPT), t-octylphenoxypolyethoxyethanol (Triton-X100), 1-chloro-2,4-dinitrobenzene (CDNB), tris hydroxymethyl aminomethane (Tris), ethylenediaminetetraacetic acid (EDTA), and H_2_O_2_ were purchased from Sigma-Aldrich (St. Louis, MO, USA). Methanol (MeOH) was acquired from Merck Life Science S.L. (Madrid, Spain). A Milli-Q water purification system (Millipore, Bedford, Burlington, MA, USA) was used to produce deionized water (resistivity < 18 MΩ cm). Standards of T-2 (MW: 466.52 g/mol), Neosolaniol (Neo; MW: 382.40 g/mol), T-2 triol (MW: 382.45 g/mol), and T-2 tetraol (MW: 298.33 g/mol) were purchased from Sigma-Aldrich (St. Louis, MO, USA). Stock solutions of the mycotoxins were made in MeOH at the proper working concentrations and kept at a constant temperature of −20 °C in the dark.

### 5.2. Cell Culture and Treatment

Human hepatocarcinoma (HepG2) cells (ATCC: HB-8065) were grown in DMEM medium with 10% NBCS, 100 U/mL penicillin, and 100 mg/mL streptomycin. A pH of 7.4, 5% CO_2_ at 37 °C, and 95% air atmosphere at constant humidity were the conditions used for incubation. In order to maintain genetic homogeneity, cells were sub-cultivated twice a week with only a small number of sub-passages. After trypsinization, the HepG2 cells were sub-cultivated in a 1:3 split ratio. The final mycotoxin concentrations assayed were obtained by adding each mycotoxin to the culture medium with a final MeOH concentration ≤1% (*v*/*v*). Controls containing the same amount of solvents were used in each experiment.

### 5.3. In Vitro Cytotoxicity

Cytotoxic effects were determined in HepG2 cells by the MTT assay. In this test, only metabolically active cells are used to evaluate whether a cell is viable by reducing soluble yellow tetrazolium salt to an insoluble purple formazan crystal through a mitochondrial-dependent reaction. The MTT viability assay was carried out as described by Ruiz et al. [38]. Briefly, at a density of 2 × 10^4^ cells/well, the HepG2 cells were seeded in 96-well tissue culture plates. After cells reached 80% of confluence, serial dilutions of T-2, Neo, T2-triol, and T2-tetraol in a fresh medium were added. Mycotoxin concentrations ranged from: 12.5 to 100 nM for T-2, 11 to 164 nM for Neo, 164 to 2620 nM for T2-triol, and 209 to 3350 nM for T2-tetraol. The range of concentrations tested for each mycotoxin was chosen based on assays done previously with all the mycotoxins. The mycotoxin was exposed for 24 h. After that, 200 μL of fresh medium was added to each well after the medium was removed. Then, 50 μL/well of MTT was added, and the plates were put back into the incubator in the dark. The MTT solution was removed after 3 h of incubation, and 200 μL of DMSO and 25 μL Sorensen’s glycine buffer were added. In order to achieve complete dissolution, plates were shaken for 5 min. Using an automatic ELISA plate reader (MultiSkanEX, Thermo Scientific, Walthman, MA, USA), the absorbance was determined at 540 nm.

Cell viability was expressed as a percentage relative to the control solvent (1% MeOH). Determinations were performed in three independent experiments. The mean inhibition concentration (IC_50_) values were obtained using SigmaPlot version 11 (Systat Software Inc., GmbH, Düsseldorf, Germany).

### 5.4. Intracellular ROS Generation

Adding H_2_-DCFDA allowed researchers to observe intracellular ROS generation in HepG2 cells after 24 h. Intracellular esterases deacetylate the H_2_-DCFDA after it has been absorbed by cells, and the non-fluorescent 2′, 7′-dichlorodihydrofluorescein (H_2_-DCF) that results is converted by ROS into the highly fluorescent dichlorofluorescein (DCF). Briefly, a 96-well black culture microplate was seeded with 2 × 10^4^ cells/well. After cells reached 80% of confluence, the culture medium was changed to fresh medium containing different concentrations of T-2 (7.5, 15, 30 nM), Neo (12.5, 25, 50 nM), T2-triol (0.12, 0.25, 0.45 µM) and T2-tetraol (0.45, 0.9, 1.70 µM) for 24 h of incubation. The range of concentrations tested for each mycotoxin was chosen in accordance with earlier research done in our laboratory based on the IC_50_ values and considering the best concentration range for each mycotoxin IC_50_/2, IC_50_/4, and IC_50_/8 values [9]. Afterward, the culture medium was removed, and cells were incubated with 50 μM H_2_-DCFDA/well in the fresh medium for 30 min. Later, the H_2_-DCFDA was removed, and cells were washed with PBS after adding 200 μL PBS/well. Increases in fluorescence were measured on a Wallace Victor2, model 1420 multilabel counter (PerkinElmer, Turku, Finland) at excitation/emission wavelengths of 485/535 nm. Results are expressed as increases in fluorescence with respect to solvent control. Determinations were performed in two independent experiments with 12 replicates each.

### 5.5. GSH Determination

The determination of GSH and GSSG was evaluated according to Maran et al. [39]. Briefly, a six-well plate was seeded with 2.25 × 10^5^ cells/well. The cells were exposed to different treatments: (a) fresh medium (DMEM medium), (b) NAC pre-treatment (DMEM medium plus 1 mM NAC), and (c) BSO pre-treatment (DMEM medium plus 60 μM BSO). Once the cells reached 80% confluence, the culture medium was replaced with fresh medium containing different concentrations of T-2 (7.5, 15, 30 nM), Neo (12.5, 25, 50 nM), T2-triol (0.12, 0.25, 0.45 µM) and T2-tetraol (0.45, 0.9, 1.70 µM) incubated for 24 h. Following removal of the medium, the cells were washed with PBS and homogenized in 0.25 mL of 20 mM Tris and 0.1% Triton. The microplate reader Wallace Victor^2^, model 1420 multilabel counter (PerkinElmer, Turku, Finland) with excitation and emission wavelength of 345 and 424 nm, was used to measure the concentrations of GSH and GSSG, respectively. The level of GSH and GSSG was represented in μg/mg proteins. Determinations were performed in three independent experiments.

### 5.6. Determination of Enzymatic Activities

In order to determine the scavenging procedures in HepG2 cells exposed to T-2 and its metabolites, the GST, GPx, CAT, and SOD activities were determined at the concentrations previously selected. For these assays, 2.25 × 10^5^ cells/well were seeded in six-well plates. The cells were exposed to different treatments: (a) fresh medium (DMEM medium), (b) NAC pre-treatment (DMEM medium plus 1 mM NAC), and (c) BSO pre-treatment (DMEM medium plus 60 μM BSO). After cells achieved the 80% confluence, cells were treated with the T-2, Neo, T2-triol, and T2-tetraol for 24 h. Then, cells were homogenized in 0.1 M phosphate buffer pH 7.5 with 2 mM EDTA after the medium was eliminated. All the enzyme determinations were performed in triplicate.

Following the conjugation of GSH with CDNB for 5 min, the GST activity was assessed using the method of [39]. The reaction mixture contained in a final volume of 1 mL: 100 μL of 20 mM GSH, 825 μL of 0.1 M Na/K phosphate buffer at pH 6.5, 25 μL of 50 mM CDNB dissolved in ethanol, and 50 μL of homogenized cell sample. The GST activity was expressed as mol of product formed/min/mg of protein using a molar absorptivity of CDNB (9.6 mM^−1^ cm^−1^). Enzymatic activity was evaluated in a thermocirculator of PerkinElmer UV/vis spectrometer Lambda 2 version 5.1 (PerkinElmer, Turku, Finland). The absorbance was measured at 340 nm.

According to Maran et al. [39], the GPx activity was measured spectrophotometrically utilizing H_2_O_2_ as a substrate for Se-dependent peroxidase activity of GPx by following oxidation of NADPH during the first five min in a coupled reaction with GR, as described by Maran et al. [39]. In 1 mL final volume, the reaction mixture contained 4 mM NaN_3_ with 500 μL of 0.1 M phosphate buffer, pH 7.5 and 2 mM EDTA, 250 μL of ultrapure water, 100 μL of 20 mM GSH, 2U GR, 50 μL of 5 mM H_2_O_2_ and 20 μL of 10 mM NADPH. Fifty microliters of homogenized cell samples were added to the reaction mixture. At pH 7.5, one unit of GPx will reduce 1 μmol of GSSG per min. Using a molar absorptivity of NADPH (6.22 mM^−1^ cm^−1^), the enzymatic activity of the GPx enzyme was determined and expressed as μmol of NADPH oxidized/min/mg of protein. Assays were carried out at 25 °C in a thermocirculator of PerkinElmer UV/vis spectrometer Lambda 2 version 5.1 (PerkinElmer, Turku, Finland). At 340 nM, the absorbance was measured.

According to Zingales et al. [17], the CAT activity was measured. Briefly, 100 μL of homogenized cell sample were combined with 400 μL of 40 mM H_2_O_2_ and 500 μL of 0.5 M potassium phosphate buffer at pH 7.2. Using a spectrophotometer (Super Aquarius CECIL 9500 CE, Milton Technical Center, Cambridge, United Kingdom), the rate of enzymatic decomposition of H_2_O_2_ was measured as decreases in absorbance at 240 nm for 3 min at 30 °C. The molar absorptivity of H_2_O_2_ (43.6 mM^−1^ cm^−1^) was used to calculate the CAT activity, which is represented as μmol H_2_O_2_/min/mg of protein.

The Ransod kit (Randox Laboratories Ltd., Antrim, United Kingdom) modified for 1.5 mL cuvettes was used to measure the SOD activity. The free radical superoxide is destroyed by the SOD by being changed into peroxide. A spectrophotometer of PerkinElmer UV/Vis Lambda 2 version 5.1 (PerkinElmer, Turku, Finland) was used to measure the SOD activity at 505 nm for 3 min at 37 °C. Units of SOD/mg protein were used to express the SOD values.

### 5.7. Determination of Protein Content

The Bio-Rad DC Protein Assay (Bio-Rad, Hercules, CA, USA), with the catalog number 5000116, was used to determine the protein content. Using an automatic ELISA plate reader (MultiSkanEX, Labsystem, Helsinki, Finland), the concentration of protein (µg/mL) was measured at 690 nm.

### 5.8. Pre-Treatment with BSO or NAC

Cells were pre-treated with NAC or BSO prior to the mycotoxin exposure to examine the effects of these compounds on the modulation of intracellular GSH content and on the enzymatic activities related to GSH content. Approximately 2.25 × 10^5^ cells/well were exposed to 1 mM NAC or 60 μM BSO dissolved in the medium for 24 h. After that, the cells were treated with fresh medium containing T-2, Neo, T2-triol, and T2-tetraol at the designated concentrations for 24 h. As a control, cells with 1% MeOH in the medium were used. The GSH content and enzymatic activities were evaluated after 24 h of exposure, as previously mentioned. Comparisons between cells exposed to different concentrations of each mycotoxin in fresh medium and NAC or BSO pre-treatment were performed.

### 5.9. Statistical Analysis

Data from various independent experiments were expressed as mean ± SEM. Student’s *t*-test for paired samples was used for statistical analysis of results. One-way ANOVA followed by the Turkey HDS *posthoc* test for multiple comparisons was used to analyze the difference between groups. A difference level of *p ≤* 0.05 was considered statistically significant.

## Data Availability

Data is contained within the article.
